# Predictors of Venous Thromboembolic Events Associated with Central Venous Port Insertion in Cancer Patients

**DOI:** 10.1155/2014/743181

**Published:** 2014-02-09

**Authors:** Christine Hohl Moinat, Daniel Périard, Adrienne Grueber, Daniel Hayoz, Jean-Luc Magnin, Pascal André, Marc Kung, Daniel C. Betticher

**Affiliations:** ^1^Department of Medical Oncology, Hôpital Cantonal de Fribourg, Chemin des Pensionnats 2, 1700 Fribourg, Switzerland; ^2^Department of Angiology, Hôpital Cantonal de Fribourg, Chemin des Pensionnats 2, 1700 Fribourg, Switzerland; ^3^Central Laboratory, Hôpital Cantonal de Fribourg, Chemin des Pensionnats 2, 1700 Fribourg, Switzerland; ^4^Pharmacy Unit, Hôpital Cantonal de Fribourg, Chemin des Pensionnats 2, 1700 Fribourg, Switzerland

## Abstract

Insertion of central venous port (CVP) catheter in the cancer population is associated with increased incidence of venous thromboembolic events (VTE). However, trials have shown limited benefit of antithrombotic treatment to prevent catheter-related venous thrombosis. This prospective observational cohort study was designed to assess the incidence of VTE closely related to CVP implantation in patients with cancer and undergoing chemotherapy, and to identify a high risk subgroup of patients. Between February 2006 and December 2011, 1097 consecutive cancer patients with first CVP implantation were included. Catheter-related VTE were defined as deep venous thrombosis in the arm, with or without pulmonary embolism (PE), or isolated PE. The incidence of CVP-associated VTE was 5.9% (IC95 4.4–7.3%) at 3 months, and 11.3% (IC95 9.4–13.2%) at 12 months. The incidence of any VTE was 7.6% (IC95 6.0–9.3%) at 3 months, and 15.3% (IC95 13.1–17.6%) at 12 months. High Khorana risk score and lung cancer were significant predictors of 3 month VTE. In conclusion, this large cohort study of patients with first CVP catheter implantation confirms the high incidence of VTE associated with the CVP implantation and allow identifying high risk patients who may benefit from thromboprophylaxis.

## 1. Introduction

The majority of patients with cancer undergoing chemotherapy require an efficient venous access for several weeks or months. Central venous port (CVP) catheter is widely used in this setting. The incidence of deep venous thrombosis (DVT) and pulmonary embolism (PE) associated with central venous catheter has been reported between 2% and 67% [1]. The cancer population combines nonspecific thromboembolic risk factors (age, malignancy, hypercoagulability, chemotherapy, infections, and bed rest) [2] and specific risk factors such as catheter material, multiple placement attempts, catheter size and length, number of lumens, and catheter tip localization [3–5]. Catheter-related VTE may be limited to asymptomatic radiological findings but may also lead to significant clinical burden with upper limb postthrombotic syndrome reported in 5 to 28% [6, 7] and respiratory failure in case of pulmonary embolism. Moreover, catheter thrombosis can also lead to catheter occlusion in 14 to 36% and delay chemotherapy [8].

At least eight randomized controlled trials have evaluated antithrombotic therapy versus placebo in the prevention of central venous catheter-associated thrombosis [9–16]. A small study found that fixed dose of warfarin 1 mg once daily reduced the incidence of upper extremity DVT at the 90th day of venography [9]. However, two subsequent trials failed to confirm any benefit with this regimen [10, 11]. In two large studies, the administration of a prophylactic dose of low molecular weight heparin (LMWH) during at least 6 weeks after the catheter insertion did not reduce significantly the incidence of upper limb DVT compared to placebo [14, 15]. A systematic thromboprophylaxis is therefore not recommended at the time of CVP implantation, and should be considered only for patients with solid tumor and additional risk factors for VTE and low bleeding risk [17]. During the last years, Khorana and colleagues developed and validated a predictive model for chemotherapy-associated thrombosis [18, 19]. This model allows identification of patients at high risk who may benefit from antithrombotic treatment during chemotherapy. To the best of our knowledge, there is no risk score available to evaluate the risk of VTE following CVP insertion and the Khorana score has not been validated in this setting. The purpose of this study was to evaluate the incidence of VTE closely associated with the insertion and use of CVP catheters and to identify high-risk patients amenable to benefit from a short course of thromboprophylaxis after CVP implantation.

## 2. Patients and Method

### 2.1. Patient Inclusion

From February 2006 to December 2011, all consecutive adult patients suffering from cancer and who were implanted with a CVP in the Surgery Department of the Cantonal Hospital, Fribourg, Switzerland (Tertiary Care Center) were screened for inclusion in this prospective cohort. We included only patients older than 18 years with first CVP implantation. All included patients were then followed up by the department of medical oncology. The study received approval from the institutional ethic committee.

### 2.2. Surgical Implantation Procedure

Implantation was performed under local anaesthesia in the operating room by dedicated surgeons. The operator always attempted to find the cephalic-subclavian junction at the right upper limb and to place a J-curved 0.035 inch guide wire in the superior cava vein. The catheter tip was then placed at the level of the thoracic rib, under fluoroscopy guidance. The chamber was then placed in the pectoral region by tunelisation from the same skin incision. When the cephalic vein was not accessible, the catheter was implanted in the subclavian vein by direct punction. There was no routine sonography or phlebography of the subclavian vein prior to the intervention except in case of known previous DVT, previous central line use or failure to pass the guide wire in a previous attempt. During the study period, the same senior vascular surgeon was in charge of the dedicated CVP surgery team. There was no ultrasound guidance during implantation. The first chemotherapy infusion was allowed at the same day of the CVP implantation. There was no systematic antithrombotic therapy administration at the time of CVP insertion.

### 2.3. Patient Follow-Up

Patients were regularly followed up clinically during chemotherapy treatment and then every 6 months after chemotherapy completion until complete remission, death of any cause, or loss of follow-up. Patients whose CVP catheter has been removed for lack of use were followed up until 6 weeks after removal. At each visit, the subjects were questioned about any local pain or upper limb swelling at the CVP side. Any other symptom suggesting upper or lower limb DVT or any thoracic symptom suggesting a PE was further investigated by compression venous sonography of the limbs or thoracic CT scan. During follow-up, patient with symptomatic anaemia received blood transfusion. Erythropoietin agent was not used in our institution. Granulocyte-colony stimulation factor administration was allowed to reduce the length of neutropenia.

### 2.4. Definition and Assessment of Outcome

The main outcome was the 3-month incidence of catheter-related VTE, defined as occlusive DVT in the arm along the catheter, with or without PE, or isolated PE of unknown origin. The secondary outcomes were the 12-month incidence of catheter-related VTE and the 3- and 12-month incidence of any VTE related or not to the catheter, including DVT of the leg, DVT of the other arm and visceral DVT. Asymptomatic DVT or PE observed on CT scan performed for tumoral staging was classified as asymptomatic event. Catheter dysfunctions were investigated by US-Doppler or phlebography. Catheter dysfunction and small, nonoccluding thrombosis along the catheter were not considered as event. Complete occlusion of the vein along the catheter was considered as asymptomatic event if it was not associated with local symptoms.

### 2.5. Khorana Score

The Khorana score is a validated tool for estimation of VTE during chemotherapy [18]. The Khorana predictive score assigns 2 points to very high-risk cancer sites (pancreatic, gastric, brain) and 1 point to high risk cancer sites (lung, ovarian, renal, or bladder). In addition, 1 point is assigned for each of the following: platelet count ≥ 350 × 10^9^/L, hemoglobin < 10 g/dL, or use of erythropoietin-stimulating agents, leukocyte count ≥ 11 × 10^9^/L, and body mass index ≥ 35 kg/m^2^. Patients with Khorana score ≥ 3 are considered at high risk for VTE.

### 2.6. Statistical Analysis

Incidences of event were expressed as proportions with 95% confidence intervals, calculated by binomial Wilson test. Proportions of event were compared using Chi^2^ test, and continuous variables were compared by the Mann-Whitney rank sum test according to the normality of their distribution. Statistical significance was considered for *α* < 0.05. The contribution of clinical characteristics (age, sex, weight, body mass index, previous VTE, respiratory failure, renal failure, antithrombotic treatment, cancer location, metastatic stage, low performance status, class of chemotherapy agent, major surgery close to CVP implantation or during follow-up, side of CVP implantation, baseline laboratory values, and Khorana score) to asymptomatic and symptomatic VTE was analysed using multivariable logistic regression analysis. Factors were first analyzed individually in univariate analysis and then selected for multivariable analysis based on a *P* value < 0.2 or known confounding effect. The statistical analysis was performed using Stata 9.0 (Statacorp, College Station, Texas, USA).

## 3. Results

### 3.1. Patients

Throughout the 6-year study period, 1243 consecutive patients were candidates for a CVP implantation in our institution and were screened for inclusion. We included 1074 patients with CVP placement at first attempt and 23 patients with failure at first attempt but success at second attempt. We excluded 146 patients (15 patients for previous CVP placement, 129 patients for choice for another intravenous access after CVP placement failure, and 2 patients for failure to place CVP at first and second attempt). The clinical characteristics of the 1097 patients included are shown in Table 1. The most frequent cancers were lung (21.1%), colo-rectal (18.6%), and oesogastric (13.3%).

### 3.2. Venous Thromboembolic Events

Table 2 shows the incidence of VTE at 3 and 12 months. The incidence of CVP-associated VTE was 5.9% of patients (IC95 4.4–7.3%) at 3 months and 11.3% (IC95 9.4–13.2%) at 12 months. The incidence of VTE at any location was 7.6% of patients (IC95 6.0–9.3%) at 3 months and 15.3% (IC95 13.1–17.6%) at 12 months.

### 3.3. Predictors of Catheter-Associated VTE at 3 Months

The multivariate logistic regression analysis identified 2 significant predictors of catheter-related VTE at 3 months: a Khorana score ≥ 3 (odd ratio (OR) 3.50, CI95 1.00 to 12.3, and *P* = 0.05) and lung cancer (OR 5.45, CI95 1.87 to 15.87, and *P* = 0.002). Low performance status was borderline significant (OR 4.68, CI95 0.97 to 22.4, and *P* = 0.054) (Table 3). Advanced stage with distant metastases was only borderline significant in the univariate analysis but fell in the multivariable regression. Previous VTE, high BMI, age > 70, and platinum-based regimen were not associated with VTE at 3 months in this cohort. The delay between CVP implantation and chemotherapy infusion was analysed. Most patients had chemotherapy close to the CVP implantation (31% of patients had chemotherapy before CVP implantation, 14.7% of patients had chemotherapy 0 to 3 days after CVP implantation, 20.6% of patients had chemotherapy 4 to 8 days after implantation, and 33.7% of patients > 8 days later). Compared to the 3 other groups, the group of patients receiving chemotherapy within days 0 to 8 after CVP implantation had no additional risk of CVP-related VTE at 3 months (OR 1.00, 0.67 to 1.57, and *P* = 0.98).

### 3.4. Incidence of VTE in High-Risk Subgroups

The incidence of VTE was particularly high in the 3 subpopulations identified. Among the 102 patients (9.3%) with a baseline Khorana score ≥ 3, 18.6% (CI95 10.9 to 26.4) had a VTE during the first 3 months of follow-up. This incidence was 10.8% (CI95 6.8 to 14.8) among the 232 patients (21.1%) with lung cancer and 10.9% (CI95 7.7 to 14.1) among the 367 patients (33.5%) with metastatic cancer at the time of CVP insertion.

### 3.5. Predictors of VTE at 12 Months

**Figure 1:**
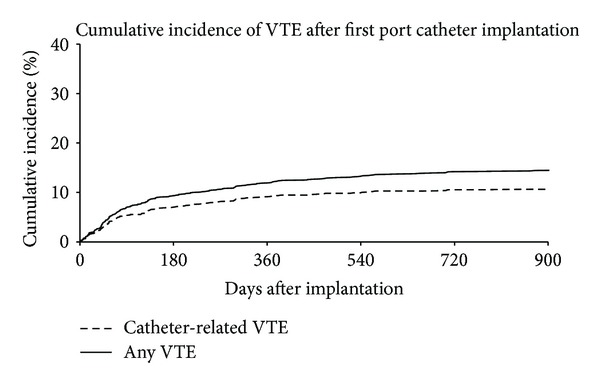


The cumulative incidence of VTE is shown in Figure 1. The steeper part of the slope is observed during the first 3 months after catheter implantation. However, additional events continue to be observed up to the end of follow-up. Multivariable regression analysis identified the same predictors of VTE at 12 months than at 3 months. Khorana score ≥ 3 (OR 2.67, CI95 1.49 to 4.78, and *P* = 0.001) and lung cancer (OR 1.93, CI95 1.15 to 3.25, *P* = 0.01) were significantly associated with VTE (from any origin) during the 12-month study period. Bevacizumab and platinol based regimen were both borderline significant predictors of 12-month VTE events (Table 3).

## 4. Discussion

This large cohort study, designed to evaluate the incidence of VTE closely related to central venous Port catheter implantation, shows that 7.6% of the patients will develop DVT or PE during the first 3 months after catheter implantation. This finding confirms the important burden linked to the IV management of chemotherapy and the importance to develop efficient preventive antithrombotic strategies. This study also validates high Khorana score and lung cancer as significant predictors of VTE during the whole study period. Despite several trials, evaluating different drug regimens to prevent chemotherapy-associated VTE, no clear benefit emerged from any specific regimen up to now [20]. One limitation of thromboprophylaxis trials is due to the fact that the benefit from antithrombotic treatment can be overwhelmed by the bleeding risk occuring during chemotherapy. The other difficulty is to identify patients at risk as well as the period at risk. Efforts have been made to identify those patients who will benefit most from thromboprophylaxis. The current practice now is to consider prophylactic antithrombotic treatment for patients with solid tumor and an additional risk factor for VTE, such as previous VTE, immobilization, and specific anticancer therapy (thalidomide or lenalidomide in association with dexamethasone) [17, 21].

Limiting thromboprophylaxis on a high-risk period could increase efficiency and reduce the bleeding risk inherent to prolonged antithrombotic treatment. The period of CVP implantation concentrates major risk factors for VTE (surgery, intravenous foreign material, upper limb immobilization, untreated cancer, and repeated chemotherapy infusions). Our study investigated specifically this period and confirmed the high incidence of VTE close to the CVP insertion and its key role in the pathogenesis of cancer-related VTE. Our study also identified 2-patient categories prone to develop VTE in 10 to 20% at 3 months and who will certainly benefit from thromboprophylaxis, at the time of CVP implantation. Interestingly, identification of these patients based on simple clinical data (lung cancer) or a widely used score based on the type of tumor, BMI, and prechemotherapy laboratory values allows easy identification of patients who could benefit from thromboprophylaxis.

In their observational study of 815 CVP implantations, Narducci et al. found that the factor most strongly predictive of complications was a delay shorter than 8 days between CVP implantation and first use [22]. These complications were mostly nonthrombotic (local of systemic infection, port expulsion, catheter dysfunction, or migration). We analyzed the delay between implantation and use in our study and found that a delay > 8 days did not reduce the 3-month VTE incidence.

One limitation of our study is that the definition of the primary outcome did not include death due to probable or possible VTE. At the time of the study design, we were concerned about being unable to exclude formally PE as the cause of death, especially in the setting of palliative care. However, we consider that the conclusions of our study are rather conservative since adding deaths for VTE origin could increase the incidence of VTE related to CVP insertion. Another limitation of our study is the lack of ultrasound guidance during CVP placement. Ultrasound guidance has shown to reduce the risk of infectious and thrombotic complications in all percutaneous venous procedures. Its role in the identification of the vein during open surgery is less clear. We think that this was probably compensated by the high experience of our surgeons. Our institution has a surgical team dedicated to CVP placement and during the study period (2006 to 2011) the same senior vascular surgeon was in charge of the team. They always tried to identify the cephalic vein for puncture. The subclavian vein was punctured only when cephalic vein was not found. In these cases, the subclavian vein was directly observed and ultrasound would not bring further security.

In conclusion, this large cohort study of consecutive patients with first CVP catheter implantation confirms the high incidence of thrombotic events closely associated with the CVP intervention. It confirms Khorana score and lung cancer as strong predictors of catheter related VTE and VTE of any origin at 3 and 12 months. These findings will allow the definition of a risk population in order to assess the best thromboprophylaxis in a randomised trial. Otherwise, we may question whether it is still reasonable to delay efficient thromboprophylaxis for these patient populations.

## Figures and Tables

**Table 1:** Clinical characteristics of the cohort including 1097 consecutive patients receiving their first implantable central venous catheter (catheter port) for antitumoral chemotherapy. *P* values are for patients with VTE (*n* = 122) versus patients without event (*n* = 933).

Clinical characteristics	All patients (*n* = 1097)	Patients without VTE (*n* = 933)	Patients with any VTE (*n* = 164)	Patients with catheter-related VTE (*n* = 122)	*P* value
Age (median, range)	62 (18 to 89)	63 (18 to 89)	62 (29 to 84)	63 (31 to 84)	NS
Age > 70 y	299 (27.3%)	261 (28%)	38 (23.2%)	26 (21.5%)	NS
Male	617 (56.2%)	527 (56.5%)	90 (54.9%)	65 (53.3%)	NS
BMI (median, range)	25.0 (14.9 to 57.8)	24.4 (14.9 to 51.4)	24.3 (16.1 to 38.1)	24.2 (16.1 to 38.1)	NS
BMI > 35	51 (4.6%)	41 (4.4%)	11 (6.7%)	10 (8.3%)	0.05
Major comorbidities					
Coronary heart disease	117 (10.7%)	108 (11.6%)	9 (5.4%)	7 (5.8%)	0.06
Diabetes	138 (12.6%)	122 (13.1%)	16 (9.8%)	15 (12.3%)	NS
Respiratory failure	306 (27.9%)	252 (27%)	54 (32.9%)	48 (39.7%)	0.002
Renal failure	63 (5.7%)	58 (6.2%)	5 (3%)	3 (2.5%)	NS
Previous VTE	118 (10.8%)	99 (10.6%)	19 (11.6%)	16 (13.2%)	NS
Cancer					
Lung	260 (23.7%)	207 (22.2%)	53 (32.3%)	47 (38.5%)	<0.001
Colorectal	206 (18.8%)	176 (18.9%)	30 (18.3%)	17 (13.9%)	0.09
Oesogastic	146 (13.3%)	128 (13.8%)	18 (11%)	14 (11.5%)	NS
Breast	120 (10.9%)	106 (11.3%)	14 (8.5%)	10 (8.2%)	NS
ORL	84 (7.7%)	76 (8.1%)	8 (4.9%)	7 (5.7%)	NS
Hepatocholangiopancreas	76 (6.9%)	64 (6.9%)	12 (7.3%)	5 (4.1%)	NS
Lymphomas	75 (6.8%)	69 (7.4%)	6 (3.7%)	5 (4.1%)	NS
Other	130 (11.8%)	107 (11.5%)	23 (14%)	17 (13.9%)	NS
Stage IV	396 (36.1%)	327 (35%)	69 (42.1%)	49 (40.2%)	NS
Performance status 2–4	629 (57.3%)	527 (56.5%)	111 (67.6%)	83 (67.7%)	0.01
CVP placed on left side	86 (7.8%)	64 (6.9%)	21 (12.8%)	17 (14.1%)	0.01
Surgery during 30 days before CVC placement	146 (13.3%)	125 (13.4%)	22 (13.3%)	15 (12.4%)	NS
Major surgery during follow-up	217 (19.8%)	180 (19.3%)	37 (22.6%)	27 (22.3%)	NS
Therapeutic antithrombotic treatment at time of CVP placement	90 (8.2%)	78 (8.3%)	12 (7.3%)	10 (8.3%)	NS
Chemotherapy, first cycle					
Platin based	510 (46.5%)	425 (45.5%)	85 (51.8%)	69 (56.6%)	0.08
Bevacizumab	34 (3.1%)	23 (3.2%)	11 (17.2%)	9 (7.3%)	0.01
Mean follow-up duration (months)	14 (13–15)	14 (13–15)	16 (13–18)	16 (13–18)	NS

VTE: venous thromboembolic event.

**Table 2:** Incidence of thrombotic events at 3 months and 12 months (proportion and CI95).

	3 months		12 months	
	All events	Symptomatic events	All events	Symptomatic events
Subclavian DVT	3.0% (1.9–4.0)	2.1% (1.2–3.1)	5.9% (4.5–7.8)	4.1% (2.7–5.4)
PE	3.0% (1.9–4.0)	1.4% (0.7–2.1)	5.5% (4.1–6.9)	2.7% (1.6–3.7)
Catheter-related event (subclavian DVT or PE)	5.9% (4.4–7.3)	3.6% (2.4–4.7)	11.3% (9.4–13.2)	6.7% (5.1–8.4)
Lower extremity or visceral DVT	2.2% (1.3–3.1)	1.3% (0.6–2.1)	4.6% (3.3–5.9)	2.8% (1.7–3.9)
All thrombotic events	7.6% (6.0–9.3)	4.7% (3.4–6.0)	15.3% (13.1–17.6)	9.3% (7.3–11.2)

**Table 3:** Predictors of VTE events during the first 3 months and 12 months following CVP catheter placement, identified by univariate and multivariate regression analysis.

	Univariate analysis or (95% CI) *P* value	Multivariate analysis or (95% CI) *P* value
3 Months catheter-related VTE event		
Lung cancer	5.3 (1.98 to 14.1) *P* = 0.0001	5.45 (1.87 to 15.87) *P* = 0.002
Khorana score ≥ 3	2.65 (0.88 to 8.0) *P* = 0.08	3.50 (1.00 to 12.30) *P* = 0.05
Low performance status	4.82 (1.08 to 21.4) *P* = 0.03	4.68 (0.97 to 22.4) *P* = 0.054
Metastatic stage	2.33 (0.86 to 6.37) *P* = 0.09	1.21 (0.41 to 3.61) *P* = 0.73
Previous VTE event	1.76 (0.54 to 5.86) *P* = 0.34	
Age > 70	0.69 (0.24 to 2.00) *P* = 0.50	
Platinum based chemotherapy	1.72 (0.66 to 4.45) *P* = 0.26	
Chemotherapy within 0 to 8 days after CVP placement	1.00 (0.62 to 1.57) *P* = 0.98	
3 months VTE event at any location		
Khorana score ≥ 3	2.59 (0.99 to 6.77) *P* = 0.05	3.56 (1.26 to 10.74) *P* = 0.01
Lung cancer	3.07 (1.37 to 6.87) *P* = 0.006	2.56 (1.06 to 6.17) *P* = 0.03
CVP inserted left	3.04 (0.89 to 10.3) *P* = 0.07	2.39 (0.64 to 8.89) *P* = 0.19
Platinum based chemotherapy	2.01 (0.90 to 4.5) *P* = 0.09	1.84 (0.74 to 4.54) *P* = 0.18
Respiratory failure	2.47 (1.11 to 5.49) *P* = 0.02	
Metastatic stage	1.96 (0.86 to 4.46) *P* = 0.11	
Low performance status	1.64 (0.67 to 4.04) *P* = 0.28	
Previous VTE event	1.42 (0.50 to 4.06) *P* = 0.50	
12 months VTE event at any location		
Khorana score ≥ 3	2.66 (1.50 to 4.70) *P* = 0.001	2.67 (1.49 to 4.78) *P* = 0.001
Lung cancer	2.16 (1.34 to 3.49) *P* = 0.002	1.93 (1.15 to 3.25) *P* = 0.01
Bevacizumab based chemotherapy	2.51 (0.89 to 7.11) *P* = 0.08	3.04 (0.98 to 9.44) *P* = 0.054
Platinum based chemotherapy	1.49 (0.93 to 2.37) *P* = 0.09	1.51 (0.89 to 3.25) *P* = 0.12
Respiratory failure	1.75 (1.09 to 2.82) *P* = 0.02	
Metastatic stage	1.49 (0.93 to 2.37) *P* = 0.17	
Low performance status	2.24 (1.33 to 3.78) *P* = 0.002	
Previous VTE event	1.35 (0.71 to 2.56) *P* = 0.35	
CVP inserted left	2.20 (1.13 to 4.26) *P* = 0.02	

## References

[1] van Rooden CJ, Tesselaar MET, Osanto S, Rosendaal FR, Huisman MV (2005). Deep vein thrombosis associated with central venous catheters—a review. *Journal of Thrombosis and Haemostasis*.

[2] Mandala M, Clerici M, Corradino I (2012). risk factors and clinical implications of venous thromboembolism in cancer patients treated within the context of phase I studies: the 'SENDO experience'. *Annals of Oncology*.

[3] Lee AYY, Levine MN, Butler G (2006). Incidence, risk factors, and outcomes of catheter-related thrombosis in adult patients with cancer. *Journal of Clinical Oncology*.

[4] Periard D, Monney P, Waeber G (2008). Randomized controlled trial of peripherally inserted central catheters vs. peripheral catheters for middle duration in-hospital intravenous therapy. *Journal of Thrombosis and Haemostasis*.

[5] Periard D (2010). Peripherally inserted central catheter in leukemia: insertion site determines clotting risk. *Leukemia and Lymphoma*.

[6] Arnhjort T, Persson LM, Rosfors S, Ludwigs U, Lärfars G (2007). Primary deep vein thrombosis in the upper limb: a retrospective study with emphasis on pathogenesis and late sequelae. *European Journal of Internal Medicine*.

[7] Elman EE, Kahn SR (2006). The post-thrombotic syndrome after upper extremity deep venous thrombosis in adults: a systematic review. *Thrombosis Research*.

[8] Baskin JL, Pui C-H, Reiss U (2009). Management of occlusion and thrombosis associated with long-term indwelling central venous catheters. *The Lancet*.

[9] Bern MM, Lokich JJ, Wallach SR (1990). Very low doses of warfarin can prevent thrombosis in central venous catheters: a randomized prospective trial. *Annals of Internal Medicine*.

[10] Heaton DC, Han DY, Inder A (2002). Minidose (1 mg) warfarin as prophylaxis for central vein catheter thrombosis. *Internal Medicine Journal*.

[11] Couban S, Goodyear M, Burnell M (2005). Randomized placebo-controlled study of low-dose warfarin for the prevention of central venous catheter-associated thrombosis in patients with cancer. *Journal of Clinical Oncology*.

[12] Abdelkefi A, Othman TB, Kammoun L (2004). Prevention of central venous line-related thrombosis by continuous infusion of low-dose unfractionated heparin, in patients with haemato-oncological disease: a randomized controlled trial. *Thrombosis and Haemostasis*.

[13] Monreal M, Alastrue A, Rull M (1996). Upper extremity deep venous thrombosis in cancer patients with venous access devices—prophylaxis with a low molecular weight heparin (Fragmin). *Thrombosis and Haemostasis*.

[14] Verso M, Agnelli G, Bertoglio S (2005). Enoxaparin for the prevention of venous thromboembolism associated with central vein catheter: a double-blind, placebo-controlled, randomized study in cancer patients. *Journal of Clinical Oncology*.

[15] Karthaus M, Kretzschmar A, Kröning H (2006). Dalteparin for prevention of catheter-related complications in cancer patients with central venous catheters: final results of a double-blind, placebo-controlled phase III trial. *Annals of Oncology*.

[16] Mismetti P, Mille D, Laporte S (2003). Low-dose molecular-weight heparin (nadroparin) and very low doses of warfarin in the prevention of upper extremity thrombosis in cancer patients with indwelling long-term central venous catheters: a pilot randomized trial. *Haematologica*.

[17] Kahn SR, Lim W, Dunn AS (2012). Prevention of VTE in nonsurgical patients: antithrombotic therapy and prevention of thrombosis, 9th ed: American College of Chest Physicians evidence-based clinical practice guidelines. *Chest*.

[18] Khorana AA, Kuderer NM, Culakova E, Lyman GH, Francis CW (2008). Development and validation of a predictive model for chemotherapy-associated thrombosis. *Blood*.

[19] Khorana AA, Connolly GC (2009). Assessing risk of venous thromboembolism in the patient with cancer. *Journal of Clinical Oncology*.

[20] Akl EA, Vasireddi SR, Gunukula S (2011). Anticoagulation for patients with cancer and central venous catheters. *Cochrane Database of Systematic Reviews*.

[21] Mandalà M, Falanga A, Roila F (2011). Management of venous thromboembolism (VTE) in cancer patients: ESMO clinical practice guidelines. *Annals of Oncology*.

[22] Narducci F, Jean-Laurent M, Boulanger L (2011). Totally implantable venous access port systems and risk factors for complications: a one-year prospective study in a cancer centre. *European Journal of Surgical Oncology*.

